# Wireless Sensor Networks Using Sub-Pixel Optical Camera Communications: Advances in Experimental Channel Evaluation †

**DOI:** 10.3390/s21082739

**Published:** 2021-04-13

**Authors:** Vicente Matus, Victor Guerra, Cristo Jurado-Verdu, Stanislav Zvanovec, Rafael Perez-Jimenez

**Affiliations:** 1 Institute for Technological Development and Innovation in Communications (IDeTIC), University of Las Palmas de Gran Canaria, 35001 Las Palmas, Spain; vguerra@idetic.eu (V.G.); cjurado@idetic.eu (C.J.-V.); rperez@idetic.eu (R.P.-J.); 2 Department of Electromagnetic Field, Faculty of Electrical Engineering, Czech Technical University in Prague, Technicka, 16627 Prague, Czech Republic; xzvanove@fel.cvut.cz

**Keywords:** optical camera communication (OCC), wireless sensor networks (WSNs), channel characterization, farming 4.0, intelligent transportation systems (ITS), visible light communication (VLC)

## Abstract

Optical wireless communications in outdoor scenarios are challenged by uncontrollable atmospheric conditions that impair the channel quality. In this paper, different optical camera communications (OCC) equipment are experimentally studied in the laboratory and the field, and a sub-pixel architecture is raised as a potential solution for outdoor wireless sensor networks (WSN) applications, considering its achievable data throughput, the spatial division of sources, and the ability of cameras to overcome the attenuation caused by different atmospheric conditions such as rain, turbulence and the presence of aerosols. Sub-pixel OCC shows particularly adequate capabilities for some of the WSN applications presented, also in terms of cost-effectiveness and scalability. The novel topology of sub-pixel projection of multiple transmitters over the receiver using small optical devices is presented as a solution using OCC that re-uses camera equipment for communication purposes on top of video-monitoring.

## 1. Introduction

Optical camera communications (OCC), the sub-field of visible light communications (VLC) in which receivers ($R_{x}$) are implemented using image sensors [1], has excellent potential to be part of the evolution of new technologies beyond the fifth generation of cellular networks (5G). The use of cameras represents a low integration cost due to their massive availability in end-user devices such as smartphones, public infrastructure surveillance cameras, and vehicular security dash cameras. Moreover, transmitters ($T_{x}$) in VLC, in general, are implemented using light-emitting diode (LED) technology, which is widely spread and presents low power consumption and a long lifespan. OCC arises from exploiting the digital cameras, considerably more abundant than individual photodiodes (PDs), but at the same time with limitations on the achievable data rates. For example, PD-based VLC systems exceeding Gbps throughput have been reported [2,3]. In contrast, the frame rate of conventional cameras poses an inherent limitation to OCC’s data rate, but the image-forming optics and the ability to visualize different objects within the field-of-view (FOV) can be exploited to increase the throughput [1,4,5,6].

OCC has been incorporated into the Institute of Electrical and Electronics Engineers (IEEE) 802.15.7r1 standard [7], which shows the interest of the scientific community in its development. The standard contemplates the two fundamental strategies for implementing these systems, which vary according to the camera’s image acquisition technique: global shutter (GS) and rolling shutter (RS) systems. The first mainly use charge-coupled device (CCD) image sensors that simultaneously expose all their pixels when acquiring a new image. On the other hand, RS systems are mainly based on complementary metal-oxide-semiconductor (CMOS) technology and scan the image sequentially line by line of pixels, with a fixed overlap [8]. Although CCD sensors are built using a GS structure, it is important to note that the image acquisition mechanism is not strictly related to the sensor’s manufacturing technology, and CMOS hardware can be built using RS or GS strategies in the readout circuitry. Moreover, OCC systems employing RS hardware can perform GS techniques for the post-processing of the image and the demodulation of data, as shown in this work.

In indoor scenarios (offices, homes, and medical or industrial facilities), VLC systems can already provide high-speed Internet, taking advantage of the short distances and the moderate presence of interfering sources. If the use of cameras is considered, one of the most prominent applications of indoor OCC is in the field of visible light positioning (VLP), which combines data transmission and image processing to recognize the geometry of the environment and monitor interactions between mobile nodes [9,10,11]. Other applications of interest have been proposed for OCC, such as wireless patient monitoring in hospitals, where the use of radiofrequency (RF) signals may interfere with the proper performance of the instrumentation and the RF spectrum is more limited than in other kinds of facilities; and peer-to-peer (P2P) data exchange using optical beacons as an alternative to near-field communications [4].

When considering the use of surveillance cameras in outdoor scenarios for Smart Cities applications and sensor networking, the presence of uncontrollable adverse atmospheric phenomena such as heat-induced turbulence, the presence of small particles in suspension (aerosols, water vapor, pollutants, dust), and rain and snow precipitation, must be taken into account. These phenomena cause light to be absorbed and dispersed, producing both attenuation and time dispersion of the signal in the link’s direction, increasing the communication error rate. Different solutions have been proposed to cope with the challenging conditions; these can be divided into ones that alter the camera’s optics and others that effectively adjust the image sensor’s internal parameters.

**Figure 1:**
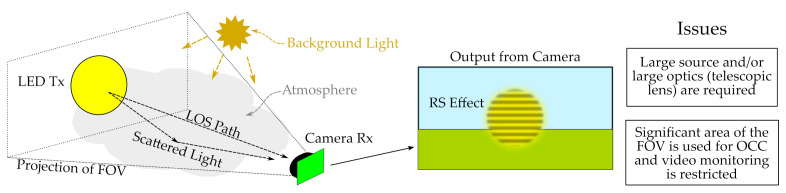
Diagram of the implementation of optical camera communication (OCC) using rolling shutter (RS) techniques in outdoor scenarios and the inherent issues associated with field-of-view (FOV) use in long distance setups.

**Figure 2:**
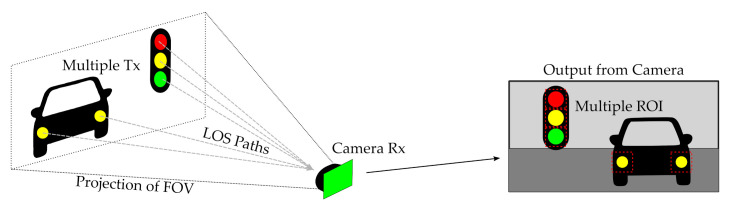
Vehicular visible light communication using OCC is an example of segregation of spatially divided data inputs that is feasible by virtue of the image-forming nature of cameras.

However, in all these works mentioned above, it is assumed that the projected area of the light source in the image affects a high number of pixels, as shown in Figure 1. In this case, the lamp’s geometric projection occupies a significant portion of the image [12,13]. This previous consideration has a final impact on the maximum distance of the link, making it necessary either to use luminous surfaces with an extensive area or to use telescopic lenses, which reduce the FOV, as mentioned before. Nonetheless, these approaches make it impossible to exploit the cameras as video and communication devices simultaneously. In contrast, the use of smaller and more numerous transmitters can be exploited in OCC for transmitting multiple data streams to the same receiver. This technique of spatial division has been proposed for intelligent transportation systems (ITS) [4] but is challenged by the need for computer vision algorithms capable of discovering the sources within the image during motion and determining the region of interest (ROI), i.e., the pixels that contain data. Figure 2 shows how multiple sources can potentially convey information to an OCC $R_{x}$ on a car.

This work extends from our previous conference paper in [14]. An OCC link under emulated fog conditions in a laboratory chamber was studied to evaluate the effects of the impairment of visibility over the process of correlation-based signal detection. The OCC camera’s output image sequences were offline processed to detect the ROI and how its dimensions varied according to the different levels of meteorological visibility produced by the presence of fog. It was shown that the correlation process was considerably affected by values under 40 m of visibility. The ROI dimensions presented a negligible change in these conditions.

Further experimentation has been carried out, including the effects of heat-induced turbulence and exploiting the camera’s analog gain in [15,16]. Real conditions of a sandstorm were experimentally studied in [17]. In this work, the previous experiments are compared and analyzed. Further theoretical analysis of the channel and the detection using correlation-based processing for ROI detection is shown in this paper.

This work’s main contribution is to demonstrate the feasibility of outdoor OCC links, in which communication happens from using not just large optical devices but also to employing small optical devices in long distances, falling into the sub-pixel level. The term sub-pixel refers to the fact that the source’s projection area is smaller than the area of one pixel, and it should not be confused with the color-sensitive components of an image sensor pixel, also called subpixels [18]. In a preliminary discussion, it could be assumed that the link is not viable because the light emitted only impinges partly one pixel. However, this work shows that the energy emitted by the LED affects the adjacent pixels for reasons ranging from the scattering in the atmosphere, and the optical focus, so that the signal can be successfully recovered by considering a larger number of pixels. This paper provides a comprehensive compilation of the authors’ highlighted findings in evaluating experimental outdoor OCC. It proposes the sub-pixel approach, discussing the viability of OCC’s real outdoor applications in the IoT and WSN fields. The OCC devices presented in this paper have been implemented using off-the-shelf components, and their designs are available for replication.

This paper is structured as follows. Section 2 summarizes the scientific contributions towards implementing optical IoT and WSN using outdoor OCC systems. In Section 3, a proposal of an architecture of OCC-based sensor networks is developed, according to the WSN key requirements and OCC capabilities. Section 4 outlines the methodology and results of the experimental evaluations done and the implications of the results obtained. Conclusions and future lines are addressed in Section 5.

## 2. Related Works

In this section, the applications and challenges of OCC and related systems are summarized, focusing on the fact that most OWC technologies, including PD-based and camera-based VLC systems, have been proposed for outdoor Smart Cities, and WSN applications [1,2,19], although some important indoor applications are mentioned as well. Specifically, regarding the implementation of sub-pixel systems for WSN as proposed in this paper, the authors are not aware of other works where an equivalent functionality is reported. It is worth noting that the sub-pixel scenario can be considered to be a VLC system based on individual PDs since camera pixels are based on such devices [18]. However, as will be seen in the following sections, the camera optics and light scattering provide an opportunity to enhance communication using the other contiguous pixels of the camera.

### 2.1. Spatial Division of Transmitters

Some of OCC’s advantages over single PD-based systems come from the image-forming nature of cameras that allow the spatial separation of the light sources as in [20,21,22]. This technique can substantially improve the data rates achievable by OCC systems if there is the possibility to extend the number of transmitters in space, as in the deployment of sensors.

One of the interesting applications that takes advantage of the spatial division is VLP [10,11,23,24]. These systems take advantage of the high precision achievable, of the order of tens of centimeters, compared to satellite positioning systems that can be tens of meters. VLP systems are robust in enclosed spaces and have been explored in outdoor vehicular settings [25] where more precision than provided by satellite localization means is needed. Furthermore, positioning systems relate to another important application, intelligent transportation systems (ITS), which has been proposed as an application of VLC [23,26,27,28]. ITS aim to improve road safety and the communication between vehicles and infrastructures. As mentioned before, vehicular VLC systems can exploit the spatial division of sources in OCC. This application has the potential to improve the performance of autonomous vehicles, an important sub-field of ITS where the use of machine learning techniques exploiting different sources of sensor data are used for lateral and longitudinal motion control of passenger cars [29]. Although many kinds of sensors are being studied, camera equipment is considered fundamental for capturing information from the environment in most approaches. In [25], computer vision and OCC techniques are combined to determine the position of a vehicle with errors below 20 cm.

OCC’s main limitations can be grouped in three categories: those related to the image-forming capability, where the distortions caused by the optics are a source of interference and noise, especially relevant in screen-to-camera communications [30]; issues related to the timing of capturing and the synchronization with transmission [12,13], in which the slight variations of the camera frame along with the gaps between frame acquisitions induce errors in data decodification; and the important challenges related to the discovery of nodes and their tracking in motion [31], where the time elapsed by computer vision algorithms in the detection of the ROI can have a considerable impact on the latency.

### 2.2. Atmospheric Phenomena in Optical Wireless Communication

Outdoor scenarios, where the weather and other atmospheric phenomena play an important role in the propagation of optical signals [32], have been experimentally investigated in the OWC field and recently in VLC, as summarized in [33]. In [34], an outdoor link of approximately 400 m was experimentally validated, which exploited defocusing the camera, allowing the surface of the LED to be extended and the transfer rate to be increased to 450 bps (bits per second). In the experiments of [26,35], other examples show how the channel’s effect is compensated by modifying the optical parameters, specifically by using magnification lenses. The first work focused on an application for a vehicular environment based on PDs instead of cameras, achieving a link distance of 40 m in ideal weather conditions. In [35], a GS camera was used to establish a link with a luminous sign located at a distance of 328 m with an effective transmission rate of 15 bps with a 4% error. Finally, Ref. [8] shows the use of a Fresnel lens for establishing a VLC vehicular link in a laboratory emulated environment is demonstrated. Nonetheless, these approaches are impractical if it is considered that the FOV of the receiver optics is drastically reduced. As a solution for maintaining the camera’s original FOV, in previous works [15,16] it is demonstrated that the signal attenuation produced by turbulence and fog phenomena can be overcome by increasing the analog gain of the image sensor without the need to alter the camera optics.

## 3. Proposal of Optical Camera Communication-Based Sensor Networks Architecture

In this section, the basic architecture of a WSN based on OCC is proposed. First, the wireless channel is derived for the case of the novel sub-pixel transmitter projection scheme, considering the outdoor scenario, where the presence of particles in the air causes both the absorption and scattering of light. A discussion of heat-induced turbulence models found in the literature is done further in this section, and it is finished by summarizing the technical requirements and potential applications of the OCC-based WSN proposed.

### 3.1. Optical Wireless Channel

As in any OWC system, the power received in an OCC system can be modeled using the solid angle differential approach (Equation (1)) [36]. Since this work focuses on outdoor links, an extinction loss term $K_{ext}\left(\lambda \right)$ has been added to the medium (absorption and scattering) which depends on the wavelength λ [37,38,39]:

(1) $$P_{rx}=P_{tx}R\left(\theta ,\varphi \right)\frac{A_{lens}}{d^{2}}\cos\left(\Psi \right)e^{-K_{ext}\left(\lambda \right)d}$$

where $P_{rx}$ is the received power, $P_{tx}$ the transmitted power and R(θ,φ) is the radiation pattern of the source (assumed constant over its entire area) for the elevation θ and azimuth φ angles. The received power depends on the area of the main lens $A_{lens}$ projected over the angle of incidence Ψ and the link distance *d*.

Nevertheless, since cameras are used as optical receivers, image-forming optics must be considered in this type of system. In general, terms and disregarding any blurring effect, a priori, OCC systems have been based on conserving pixel energy density with distance, i.e., the energy of each pixel has no direct dependence on *d*, as long as the optical emitter’s projection is greater than a single pixel, as derived in [40]:

(2) $$H_{p}\left(0\right)=\frac{A_{px}^{2}A_{lens}}{f^{2}A_{tx}}R\left(\theta ,\varphi \right),$$

where *f* is the focal length of the lens of the camera, and $A_{px}$ and $A_{tx}$ are the area of a pixel of the image sensor and the transmitter LED, respectively. The concept behind this property is the compensation of spherical propagation losses with the focus of the image. Although less energy reaches the camera’s main optics as the distance increases (quadratic decrease), the number of pixels on which the emitter is projected also decreases in the same order, compensating for the effects and keeping the surface energy density constant on the image sensor.

For long distances, it is easy to demonstrate that the number of pixels $N_{px}$ on which an emitter with area $A_{tx}$ is projected depends on the camera’s FOV and image sensor resolution as:

(3) $$N_{px}=\frac{NM}{FOV_{n}FOV_{m}}\frac{A_{tx}}{d^{2}},$$

where *N* and *M* define the horizontal and vertical resolution of the sensor, respectively, and $FOV_{n}$ and $FOV_{m}$ define the horizontal and vertical fields of vision of the receiver, respectively. By joining Equations (1) and (3), and projecting the energy over the area of a pixel, Equation (4) is obtained, which summarizes the average energy of a pixel:

(4) $$P_{px}=P_{tx}R\left(\theta ,\varphi \right)\frac{A_{lens}}{A_{tx}}\cos\left(\Psi \right)e^{-K_{ext}\left(\lambda \right)d}\zeta_{xy}A_{px},$$

where for convenience, the angular resolution of the sensor $\zeta_{xy}$ represents the ratio of its FOV to the sensor resolution. However, when the emitter’s projection decreases below a single pixel, the above equation is no longer valid and the system can start to be modeled as a PD-based OWC link, where the received power is directly proportional to the photodiode area illuminated by the projection (this projection is less than $A_{px}$ and no image can be formed). The power received in a sub-pixel situation ($P_{subpx}$) is given by:

(5) $$P_{subpx}=P_{px}\times N_{px}=P_{rx}\times A_{px}.$$

It must be understood that $N_{px}$ (the number of pixels of the transmitter’s projection) in the equation above acts as a coefficient of adjustment referred to the percentage of illuminated pixels. It is clear that $P_{subpx}$ loses its independence from distance and starts behaving as in case of a traditional OWC link. Once the arrival power to the sensor is specified, the conversion process must be taken into account when describing the OCC signal within the captured image. CMOS cameras work by converting the incident photons into electrons, storing them and encoding them sequentially row by row [18]. Therefore, it is convenient to carry out a unit conversion that takes into account this particularity. The number of stored electrons ($E_{px}$) during the exposure time of the capture $T_{exp}$ (valid for any situation) is obtained by [41]:

(6) $$E_{px}=T_{exp}\int_{\lambda }P_{px}\left(\lambda \right)EQE\left(\lambda \right)\frac{E_{ph}\left(\lambda \right)}{q}\;d\lambda$$

Please note that the concept of pixel arrival power has been extended to include the emission spectrum of the optical source. EQE(λ) is the external quantum efficiency of the receiver substrate (usually silicon), $E_{ph}\left(\lambda \right)$ is the energy of the photon at each wavelength, and *q* is the charge of the electron. Although theoretically, a small emitter located at a long distance will appear as a single bright spot in the capture, the image-forming optics are not perfect, and there is some spatial dispersion of the energy. This dispersion is modeled by the point spread function (PSF) of the system, denoted as h[n,m] already in the image domain, where *n* denotes the horizontal coordinate, and *m* the vertical coordinate. In essence, h[n,m] is the system’s spatial impulse response and is usually dependent on the distance of the link. Any projection must be convoluted with it, so in a sub-pixel situation, the illuminated region can be modeled as:

(7) $$s\left[n,m\right]=G_{V}K\left(E_{px}\right)\times h\left[n-n_{0},m-m_{0}\right]$$

where K(×) is a function that includes analog-to-digital conversion, $G_{V}$ is the analog gain of the CMOS camera’s reading circuitry and $n_{0}$ and $m_{0}$ are the coordinates of the pixels where the emitter is projected. It must be noted that in an ideal situation h[n,m] has unit energy, so if there is energy dispersion, the theoretical pixel level of the projection will be lower than expected.

Regarding the signal-to-noise ratio (SNR) of an OCC link, Equation (8) summarizes it, being applicable to both sub-pixel and generally studied situations, as:

(8) $$SNR=\frac{G_{V}^{2}E_{px}^{2}}{G_{V}^{2}\left(\sigma_{sh}^{2}+\sigma_{th}^{2}\right)+\sigma_{adc}^{2}}.$$

It has been assumed that the correction factor γ[18] of the camera is unitary for simplicity and without loss of generality, as well as that the link is not saturated (number of stored electrons less than the full-well capacity of the circuitry). The three main contributions to the noise of the OCC link are shot noise ($\sigma_{sh}^{2}$), thermal noise ($\sigma_{th}^{2}$) and quantization noise ($\sigma_{adc}^{2}$). The effect of the latter can be minimized by applying the optimal analog gain value, as demonstrated in [15]. Among the noises of shot nature, the most significant contributions are the dark noise, the shot noise of the generated signal itself, and the readout noise. In outdoor links there is another phenomenon that can have a substantial impact on system performance. The background level can vary, at least in a sub-pixel situation where the speed is determined by the camera’s capture rate, comparable to the transmission time of a frame. This effect will be analyzed experimentally in Section 4.

As derived in [15,42,43], turbulence is a consequence of the heterogeneous values of temperature and pressure in the atmosphere. The refractive index of the air changes randomly over time and space, affecting the amplitude and phase of optical signals [36]. The refractive-index structure parameter ($C_{n}^{2}$) (in m${}^{-2/3}$), is used to characterize the strength of optical turbulence. It typically ranges from 10${}^{-17}$ m${}^{-2/3}$ or less for weak turbulence, and above 10${}^{-13}$ m${}^{-2/3}$ for strong turbulence. It is given by [43,44,45]:

(9) $$C_{n}^{2}={\left(79\times 10^{-6}\frac{P}{T^{2}}\right)}^{2}\times C_{T}^{2},$$

where *P* and *T* are the average values of the air’s pressure in millibar and temperature in Kelvin, respectively. $C_{T}^{2}$ is the temperature structure parameter, which can be calculated by measuring the temperature of two or more points in the space separated by a distance *R*. It is derived from the random processes’ general definition of the structure function $D_{T}$, given by [45]:

(10) $$D_{T}=\langle {\left(T_{1}-T_{2}\right)}^{2}\rangle =\left\{\begin{array}{cc} C_{T}^{2}\times l_{0}^{-4/3}\times R^{2} & 0\ll R\ll l_{0}\\ C_{T}^{2}\times R^{2/3} & l_{0}\ll R\ll L_{0} \end{array}\right.,$$

where $|T_{1}-T_{2}|$ is the temperature difference between two points, and $l_{0}$ is the minimum air heterogeneity characterized by Kolmogorov’s theory of turbulence [45], whereas $L_{0}$ is the maximum.

### 3.2. Technical Requirements and Potential Applications of OCC-Based Wireless Sensor Networks

**Figure 3:**
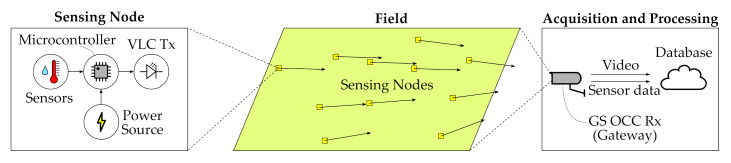
Proposal of the OCC equipment for Wireless Sensor Networks.

As mentioned before, OCC has many potential applications in different scenarios. This technology is cost-effective and allows simultaneous communication with a significant number of remote nodes, providing dedicated time-frequency channels to each of them thanks to optical cameras’ inherent spatial division multiplexing capabilities. A general-purpose scheme of an OCC-based WSN has been defined and it is depicted in Figure 3. This baseline description includes simple low power receiver-less remote nodes, the deployment scenario, the camera-based gateway, and a cloud-based endpoint. Depending on the use case characteristics, the sensors’ information may be extracted on-the-edge by processing the captured frames in situ (at the camera side) or processing them in the cloud endpoint after streaming the captured video signal. Some remarkable application fields have been identified and are discussed in the following subsections.

IoT technology is beginning to impact the agriculture industry, providing unforeseen capabilities that comprise, among others, local or remote data acquisition, communication between critical agents, and cloud-based intelligent decision making. These capabilities are expected to improve not only the yields but also optimize essential resources such as land and water and even help the workforce. According to [46], the main applications of Smart Agriculture are the monitoring of water, soil, bugs, crop health, machinery, and the environment. These applications rely on several services such as irrigation, fertilization, or soil preparation, which ultimately make intensive use of sensors that have particular connectivity demands.

Communication in Farm Area Networks (FANs) is being carried out using the available cellular infrastructures, IEEE 802.15.4-based technologies such as Bluetoothor Zigbee, LoRa, or Sigfox. Regarding the use of 2G-4G technology, the availability of these deployments is a primary concern in rural areas, and the use of Low Power Wide Area Network (LPWAN) technologies such as LoRa [47], or Sigfox [48] are mainly being adopted in the industry. This communications layer is usually the lowest level of a four-layer architecture, including Medium Access Layer (MAC), Network Layer, and Transport Layer. Notwithstanding, following the proposed scheme of Figure 3, an OCC-enabled FAN using sub-pixel links would need only a physical layer implementation in the first mile, while the camera would act as a data-aggregating agent which could have traditional interfaces such as the mentioned ones. The advantages of integrating OCC in this use case are the unlicensed spectrum, the potential capability of simultaneously monitoring hundreds of devices without MAC protocol, and the simplicity of the node design.

## 4. Experimental Evaluation

In this section, the experimentation using different OCC equipment is detailed by describing their key parameters and modulation scheme and presenting the experimental setups and the results obtained in various realistic scenarios.

### 4.1. Physical Layer Strategies

**Figure 4:**
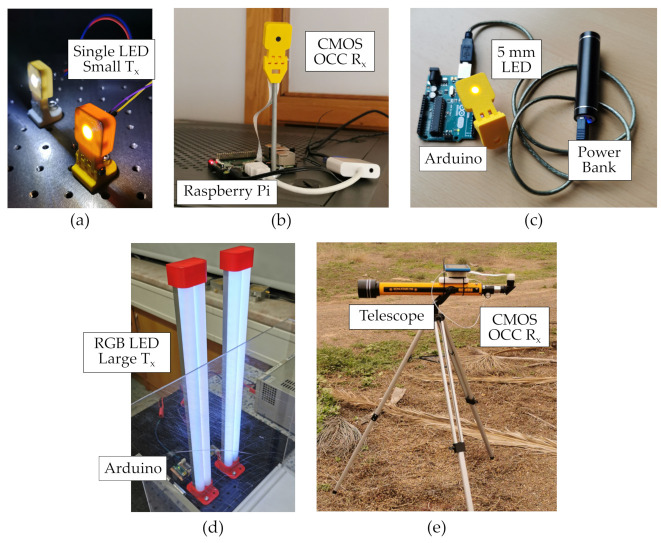
Hardware and equipment developed for the OCC experiments. (**a**) Single LED small transmitters. (**b**) CMOS camera-based OCC receiver. (**c**) Standalone implementation of the single LED small transmitters. (**d**) RGB LED large transmitters. (**e**) Large optics (telescope) CMOS-based receiver.

The transmitters and receivers developed for the experiments shown in this section consist of LED modules and CMOS cameras with different optics, respectively. They can be separated into two categories of small and large optical devices. The small devices consist of discrete off-the-shelf components, and the large devices have been developed for an extended vertical dimension (for the case of $T_{x}$) and for a longer focal distance (for the case of $R_{x}$). The large transmitters have also been implemented using multi-channel red-green-blue (RGB) LEDs, for exploiting more parallel data streams, with a higher power consumption in consequence. Figure 4 shows the implementation of these devices, while Table 1 shows their key parameters.

**Figure 5:**
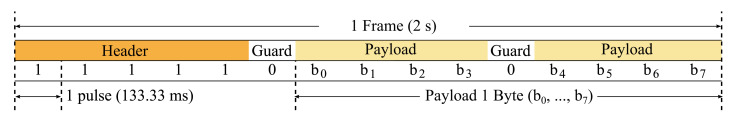
Frame format used by both of the $T_{x}$ devices of the sub-pixel experiment, assuming a global shutter demodulation at the $R_{x}$.

The modulation scheme used by the transmitters is on-off keying (OOK), and takes advantage of the switching outputs available in most microcontrollers and only requires the use of a transistor for driving high power LEDs without complex front-end devices. In the case of low power LEDs, the switching output can usually directly drive the LED. The packet structure proposed for GS detection is depicted in Figure 5, with a symbol rate of 7.5 baud for cameras using 30 fps frame rate. In the case of RS detection, much higher symbol rates can be employed, proportional to the row-shift time, as described in [13]. The RS experiment used a symbol rate of 8.4 $\times 10^{3}$ baud, and exploited the color channels of the camera, with three parallel data streams using RGB LEDs.

### 4.2. Description of the Experiments

The experiments carried out range from laboratory setups emulating outdoor scenarios [14,15,16] to real outdoor scenarios with different conditions [17], as summarized in Table 2 and described in the following sections. The OCC equipment was developed using off-the-shelf components, such as arrays of RGB LEDs in strip format and standard resin-encapsulated 5 mm white LED for the transmitters, and a RS CMOS camera with a built-in microlens. In the case of [17], the camera was attached to a telescope for covering distances up to 200 m.

#### 4.2.1. Emulation of Atmospheric Conditions in Laboratory

These experiments, labeled as 1.1 and 1.2 in Table 2, consisted of testing the large transmitters and the CMOS $R_{x}$ in laboratory settings emulating two important atmospheric conditions: fog and turbulence. First, in Experiment 1.1, only attenuation of the signal was emulated using a white methacrylate sheet in different configurations. The different optical powers received by the camera were captured changing its analog gain, and a control algorithm was derived in [16] to automatically set the gain by using the Pearson’s correlation coefficient ($r_{x,y}$) as an estimator of the image quality, alternative to the SNR. This coefficient is defined as:

(11) $$r_{xy}=\frac{\sum_{i=1}^{N}\left(x_{i}-\bar{x}\right)\left(y_{i}-\bar{y}\right)}{\sqrt{\sum_{i=1}^{N}{\left(x_{i}-\bar{x}\right)}^{2}}\sqrt{\sum_{i=1}^{N}{\left(y_{i}-\bar{y}\right)}^{2}}},$$

where $x_{i}$ are reference of *N* samples of an expected signal or template (a header of a packet, for example), $y_{i}$ are *N* consecutive samples of the input signal, and $\bar{x},\;\bar{y}$ are their mean values.

**Figure 6:**
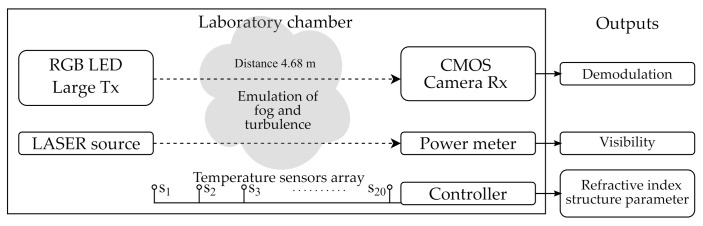
Diagram of the laboratory chamber employed for emulation of atmospheric conditions.

In the Experiment 1.2 (See Table 2), the CMOS $R_{x}$ and large $T_{x}$ (See Table 1) were tested using the laboratory chamber at the facilities of the Czech Technical University in Prague. Conditions of fog and turbulence were generated using a glycerin machine and two heater-blowers, respectively. The features of the chamber are listed in Table 3. The level of fog was studied by means of the meteorological visibility (V) [51] measured by a laser source-power meter couple aligned across the chamber, parallel to the OCC link, and the turbulence level was estimated by the well-known refractive-index structure parameter, as derived in [42], using an array of 20 temperature sensors set up equidistantly across the chamber. The diagram of the setup is shown in Figure 6.

**Figure 7:**
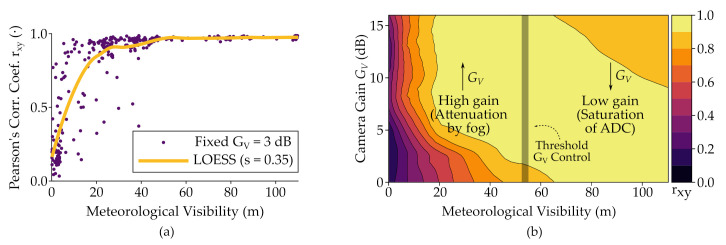
Results using the Pearson’s correlation coefficient between reference signal and images taken in Experiment 1.2 under different visibility conditions. (**a**) Scatterplot of the $r_{xy}$ values obtained using fixed gain of 3 dB. The red line is the non-parametric locally estimated scatterplot smoothing (LOESS) of span = 0.35 [52]. (**b**) Contour plot LOESS of span = 0.15 of $r_{xy}$ values changing the camera gain. The shaded vertical line corresponds to a fuzzy threshold determined by the analog gain control algorithm.

From the experimentation under heat-induced turbulence of values of $4.69\times 10^{-11}$ m${}^{-2/3}\leq C_{n}^{2}\leq 7.13\times 10^{-11}$ m${}^{-2/3}$, these conditions showed negligible influence over the OCC system performance. Contrarily, under fog conditions, the OCC system showed susceptibility to being affected by the attenuation caused by the aerosol generated by the glycerin machine. In [14] it was shown that the meteorological visibility under 40 m would make $r_{xy}$ to drop, as shown in Figure 7a. The combined effect of the low values of visibility and the low values of camera exposure cause the ADC input to be considerably low. In [15], the same visibilities were analyzed varying the camera’s analog gain, and it was shown that $G_{V}$ could overcome this issue without the need for large optics in the range of 4.68 m link distance and visibility under 40 m, which could be compared to dense fog weather. It was seen that for better visibilities, above 50 m, the gain can also cause saturation of the ADC, inducing noise. Then, between 40 and 50 m of visibility, the gain control algorithm mentioned before could use a fuzzy or adaptive threshold from which the analog gain would take high or low values, as depicted in Figure 7b.

#### 4.2.2. Real Conditions of Sandstorm Using Large Optical Devices

**Figure 8:**
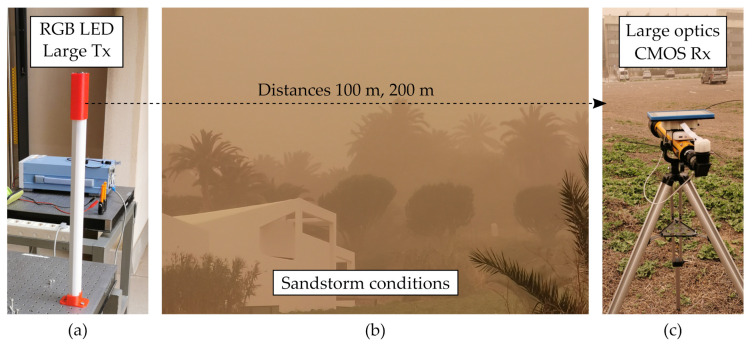
Photographs of the Experiment 3 setup under sandstorm conditions [17]. (**a**) Transmitter side using large RGB LED. (**b**) Surroundings of the experiment affected by the sand particles. (**c**) Receiver side using the CMOS camera attached to a telescope.

In the previous work, [17], Experiment 2 (See Table 2), a transmission using the large RGB LED transmitter, and the CMOS receiver attached to a 700 mm Galilean telescope was performed at distances of 100 m and 200 m during a sandstorm event in the nearby area of the IDeTIC facilities, as shown in Figure 8. The large optics used ensure a considerable area of projection over the image sensor, allowing use of RS decoding. A Gaussian Mixture Model (GMM) was used to for the accurate segregation of background and signal. It was observed that the ROI expanded in the presence of aerosols due to scattering, allowing the decoding of around 30% more lines involved in the RS detection, compared to clear conditions.

The visibility during the experiment was estimated to be about 0.57 km. The estimated $K_{ext}\left(\lambda \right)$ values for the RGB channels studied are shown in Table 4. In this high optical extinction scenario, the camera gain has a favorable effect on the SNR, improving it by up to ΔSNR≈ 9 dB at 100 m and 3 dB at 200 m. The obtained BER values for each link span are 9.14 × 10${}^{-5}$ and 4.1 × 10${}^{-3}$ for 100 m and 200 m, respectively.

#### 4.2.3. Real Outdoor Scenario in Sub-Pixel Setting

In the sub-pixel conditions of Experiment 3 (See Table 2), the small LED transmitters’ projection is less than a single camera pixel. When using image-forming optics, the emitters’ physical size is a critical aspect in establishing the links. However, at long distances, the use of emitters that are projected onto several pixels perceptible by the human eye is unfeasible since this magnitude increases quadratically with distance.

**Figure 9:**
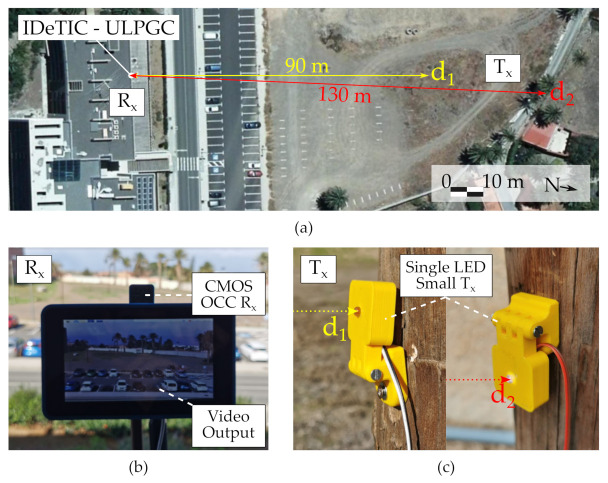
Photographs of the communication devices deployed for the sub-pixel experiments at the facilities of IDeTIC. (**a**) Satellite image from Cartográfica de Canarias (Grafcan) [53]. (**b**) CMOS camera receiver. (**c**) single LED small transmitter.

Two nodes were programmed to send OOK signals in loop transmission frames with the structure shown in Figure 5 containing 1 Byte of payload in which the values from 0x00 to 0xFF were transmitted sequentially. The bit time defined for the experiments was 133.33 ms, offering a bit rate of 4 bps. Both units were anchored to two posts located at $d_{1}$ = 90 m and $d_{2}$ = 130 m away from the camera respectively, as shown in Figure 9. Once the nodes were activated and started transmitting data, a 10-min video was recorded using 85 s of exposure time, a minimum analog gain and no digital gain. The camera’s capture rate was set to 30 frames per second, so each transmitted bit was spread over 4 frames. Once the video was captured showing the emissions of both nodes simultaneously, the regions of interest of both transmitters were defined manually, since the elaboration of a discovery procedure was not the objective of this work. Both regions of interest were statistically analyzed to obtain signal-to-noise ratio (SNR) and PSF estimates.

To carry out the SNR analysis, a 150-sample sliding window was processed in which a Gaussian Mixture Model (GMM) was set as:

(12) $$G_{2}\left(x\right)=\frac{\alpha }{\sigma_{0}\sqrt{2\pi }}e^{-\frac{{\left(x-\mu_{0}\right)}^{2}}{2\sigma_{0}^{2}}}+\frac{1-\alpha }{\sigma_{1}\sqrt{2\pi }}e^{-\frac{{\left(x-\mu_{1}\right)}^{2}}{2\sigma_{1}^{2}}}$$

where $G_{2}\left(x\right)$ is a Gaussian mixture, α is the ratio of the first Gaussian, $\mu_{i}$ denotes the expected value and $\sigma_{i}$ is the standard deviation.

The SNR is then calculated as:

(13) $$SNR=\frac{1}{2}\frac{|\mu_{1}-\mu_{0}{|}^{2}}{\alpha \sigma_{0}^{2}+\left(1-\alpha \right)\sigma_{1}^{2}}$$

The SNR of each of the samples resulting from applying the slider window was stored to estimate the system’s expected SNR afterward. Assuming that the transmission is OOK, the theoretical error rate for each experimentally estimated SNR was calculated using:

(14) $$BER=\frac{1}{2}\mathrm{erfc}\left(\sqrt{\frac{SNR}{2}}\right),$$

where erfc(×) is the complementary error function. Both SNR and BER calculations were carried out for each color of the image sensor (R, G and B) and for an average emphasized with the PSF approximation (Equation (15)). The goal of spatial averaging is to improve the SNR by reducing the effective variance of noise.

(15) $$y=\frac{\sum_{i=0}^{I-1}\sum_{j=0}^{J-1}r_{xy}\left[i,j\right]\times x\left[i,j\right]}{\sum_{i=0}^{N-1}\sum_{j=0}^{M-1}r_{xy}\left[i,j\right]},$$

where *I* and *J* are the height and width of the ROI considered for averaging, $y_{mean}$ is the signal resulting from the arithmetic mean, *y* is the signal resulting from the emphasized averaging, s[i,j] is the original signal in the coordinate (i,j).

**Figure 10:**
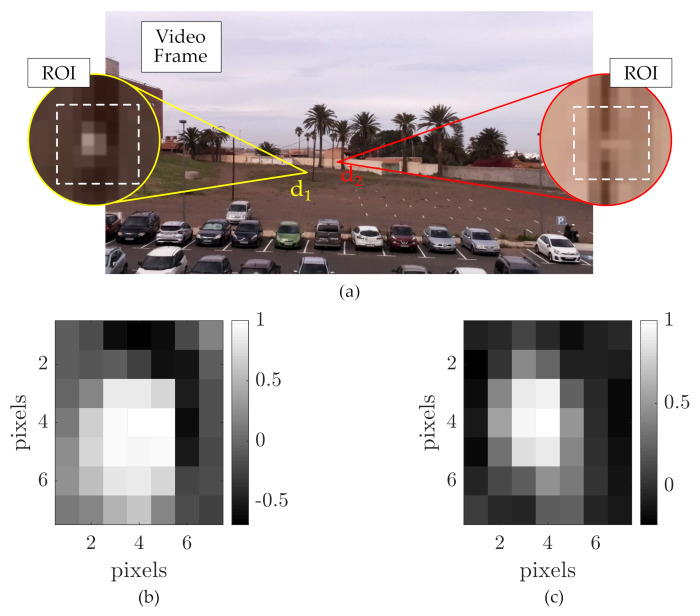
Image processing results from the experimentation using the sub-pixel setting. (**a**) Example of a frame obtained by the camera during experimentation, with insets of the regions of projection of each transmitter. (**b**) Estimated point spread function (PSF) for $d_{1}$. (**c**) Estimated PSF for $d_{2}$.

The location of the transmitters in the captured images was selected arbitrarily and reinforced by the correlation process to estimate the PSF. The image processing is depicted in Figure 10, showing the photograms obtained by the camera, and the ROI of the sources. The PSFs estimated are plotted in a region of 7-by-7 px in which it can be seen the numerous pixels that have a considerable correlation to the sub-pixel projection. The results of SNR and BER for both $T_{x}$ are summarized in Table 5. The RGB channels do not show considerable difference. The SNR at $d_{1}$ reaches 20.0 dB for the green channel, and at $d_{2}$ it is 13.0 dB for the red channel. The experimental BER values obtained do not reflect the theoretic expected values possibly due to the non-stationary behavior of the background light level, and the limited amount data analyzed.

### 4.3. Discussion of the Results

One of OCC technology’s recurring promises is the possibility of using cameras in Smart Cities for massive sensor monitoring applications. However, until now, OCC systems have been based on the use of large lamps, complemented with high-gain optics (telescopes or focal length lenses of hundreds of mm) or short distances, to make use of RS techniques. However, in the cases of use in WSN, the transmission speed is a non-critical factor, so it allows the exploitation of the inherent spatial multiplexing capacity of the cameras.

The sub-pixel system achieved a relatively equal SNR for the red, green, and blue channels, with values of approximately 20 dB and 13 dB for 90 m and 130 m, respectively, using the emphasized PSF enhancement. Considering that the NRZ-OOK technique was used, the theoretical BER level was estimated for each distance, and it can be observed that the experimental BER is considerably far for the $d_{2}$ case since the channel is non-stationary. For the case $d_{1}$, no errors were found during the experiments. The channel fluctuations are an issue for the successful segregation of the signal of interest and could become critical for mobile nodes and outdoor conditions.

## 5. Conclusions

In this paper, a network architecture based on OCC for WSN and IoT applications is developed from the advances in experimental channel evaluation in emulated and real conditions. The experimental setup used for the proof-of-concept of the network strategy was implemented using two transmitters at 90 m and 130 m communicating simultaneously to one CMOS camera at 8 bps each, with the potential to expand the number of $T_{x}$ nodes to several tens of them covering considerable areas, such as crop fields, streets, parks, industrial facilities, among others. WSNs are envisioned in this paper as a field in which sub-pixel OCC has a significant potential to become a competent alternative. Although the achievable data rate is relatively low, signaling sensor data through OCC with single LEDs is cost and energy-efficient, and camera-based receivers can be reused for video recording and communications simultaneously in these settings.

The analysis of previous works analyzed, in which RS-OCC systems were evaluated in emulated and real outdoor scenarios, showed significant limitations of RS schemes. Although the achieved data rate (several hundred bps), distances (hundreds of m), and cost-effectiveness of the equipment are positive aspects of RS-OCC, the need to use large optical devices is an issue. The use of large LED $T_{x}$ or long focal distance lenses are requirements that limit the camera equipment to only be used as a communication device at the expense of their video-monitoring capabilities. The sup-pixel approach uses only small optical devices, i.e., a microlens CMOS camera, and single 5 mm standard LEDs, allowing more transmitters to input data into the receiver, taking advantage of the spatial division that is inherent in the camera equipment, and allowing the images to be used for video-monitoring of the scenario. This re-use of camera equipment for communication and video-monitoring enables the opportunity to apply these schemes to camera systems that are already deployed, e.g., surveillance cameras.

Future works should include further experimentation in the sub-pixel scenario that supports the capabilities of the OCC-based WSN proposal envisioned in this work, including a larger number of nodes and more extended data transmissions. Other important issues to be addressed include the characterization of the channel background light in a long-term measurement, the mobility support and node discovery, which are critical for the latency of the communications. Please note that node detection in this work was done arbitrarily and offline. However, an online correlation-based detection should be implemented for automatic node discovery and for covering mobility and perturbations that might displace the transmitters’ projections on the image sensor. Furthermore, multi-hop schemes and OWC downlink from the network gateway to the sensing nodes could also be developed, although the cost of implementing optical receiver equipment in the sensing nodes can involve a high energy consumption. Finally, the experimental setup in outdoor conditions shown here can be extrapolated to small indoor environments of SCOH, and the sub-pixel context can be kept by using micro-LED devices, which are more power-efficient.

## Figures and Tables

**Table 1:** Description of the $T_{x}$ and $R_{x}$ key features.

Feature	Description
**RGB LED Large Transmitter**	
Device	12 V DC RGB LED strips (108 × 5050 SMD chips)
Front-end device	Microcontroller Atmel ATMega328p [49]
**Single LED Small Transmitter**	
Device	3.7 V DC White LED 5 mm
Front-end device	Microcontroller Atmel ATMega328p [49]
**CMOS Camera Receiver**	
Camera	Picamera V2 module (Sony IMX219 [50])
Max resolution	3280×2464 px
Gain ($G_{V}$) max. value	16 dB
Frame rate	30 fps

**Table 2:** Summary of the experiments carried out, with their key contributions on methodology and results.

Experiment	Design	Processes	Metrics	Highlighted Findings
Exp. 1.1 [16]	Attenuation emulated in laboratory. 0.46 m link.	Rolling Shutter, Gain control algorithm.	Pearson’s Corr. Coef.	Automated gain optimization.
Exp. 1.2 [14,15]	Fog and turbulence emulation in chamber, 4.68 m link.	Rolling Shutter, RGB cross-talk compensation, ROI detection.	SNR, Pearson’s Corr. Coef.	Influence of camera gain.
Exp. 2 [17]	Sandstorm real outdoor scenario. 100 m, 200 m link.	Rolling Shutter, Large optical zoom, Tilt compensation, Gaussian Mixture Model, ROI detection.	SNR, BER.	ROI expansion due to scattering.
Exp. 3	Sub-pixel real outdoor scenario. 90 m, 130 m link.	GS detection with RS hardware, Small optical devices.	SNR, BER, PSF.	Re-use, PSF enhance, Scalability.

**Table 3:** Laboratory chamber parameters and equipment.

Feature	Description
Dimensions	4.9 m (length), 0.4 m (width), 0.4 m (height)
Temperature sensors	20 × Papouch Corp. TQS3-E (precision 0.1${}^{\circ }$ C)
LASER source	Thorlabs HLS635 (635 nm) F810APC
Optical power meter	Thorlabs PM100D S120C
Heat blowers	2 × Sencor SFH7010, 2000 W
Fog machine	Antari F-80Z, 700 W

**Table 4:** Extinction coefficient values under sandstorm measurements.

Channel	λ [nm]	$K_{\mathrm{ext}}\left(\lambda \right)$ [m${}^{-1}$]
Red	630	$7.2\times 10^{-3}$
Green	530	$6.9\times 10^{-3}$
Blue	475	$5.6\times 10^{-3}$

**Table 5:** Signal-to-noise (SNR) ratio and bit error rate (BER) results by channel using point spread function weighted scheme in sub-pixel experiments.

Metric	Position	Channel R	Channel G	Channel B
SNR (experimental)	$d_{1}$	19.7 dB	20.0 dB	19.8 dB
	$d_{2}$	13.0 dB	12.9 dB	12.5 dB
BER (theoretical)	$d_{1}$	<$10^{-12}$	<$10^{-12}$	<$10^{-12}$
	$d_{2}$	$3.97\times 10^{-6}$	$5.03\times 10^{-6}$	$1.24\times 10^{-5}$
BER (experimental)	$d_{1}$	<$3.33\times 10^{-3}$	<$3.33\times 10^{-3}$	<$3.33\times 10^{-3}$
	$d_{2}$	$9.60\times 10^{-3}$	$9.60\times 10^{-3}$	$7.20\times 10^{-3}$
